# Occupational resource profiles for an addressee orientation in occupational health management: a segmentation analysis

**DOI:** 10.3389/fpsyg.2023.1200798

**Published:** 2023-07-20

**Authors:** Julian Friedrich, Anne-Kristin Münch, Ansgar Thiel, Susanne Voelter-Mahlknecht, Gorden Sudeck

**Affiliations:** ^1^Faculty of Economics and Social Sciences, Institute of Sports Science, University of Tübingen, Tübingen, Germany; ^2^Institute for Clinical Epidemiology and Applied Biometry, University Hospital and Faculty of Medicine University of Tübingen, Tübingen, Germany; ^3^Institute of Occupational Medicine, Charité—Universitätsmedizin Berlin, Freie Universität, Berlin and Humboldt-Universität zu Berlin, Berlin, Germany

**Keywords:** resource profiles, job demands and resources, addressee orientation, latent profile analysis, occupational health literacy, occupational health management

## Abstract

**Introduction:**

In order to make sustainable decisions in precision prevention and health promotion, it is important to adequately assess people's demands and resources at work. To reach them in an addressee-oriented way, a segmentation of employers and employees based on occupational resources is a promising option. We identified profiles based on personal and perceived organizational resources. Furthermore, we used job demands for profile descriptions to obtain a deeper understanding of the profiles, characterizing people with similar occupational resources.

**Methods:**

Personal occupational resources (occupational health literacy and self-efficacy) and perceived organizational resources (job decision latitude and participation in health at work) were assessed among employers and employees (*n* = 828) in small- and medium-sized enterprises in Germany. Job demands, socioeconomic status, and hierarchy levels in the company were used for further profile descriptions.

**Results:**

A six-profile solution fitted best to the data based on cluster and profile analyses. One profile was characterized by above-average occupational resources, and another profile was characterized by below-average resources. The other four profiles showed that the individual and perceived organizational resources contrasted. Either organizational resources such as job decision latitude existed and personal resources were not highly developed or people had high individual motivation but few possibilities to participate in health at work. People with medium or high job demands as well as people with low socioeconomic status were most frequently in below-average resource profiles. Employers with high hierarchy levels were overrepresented in the above-average profiles with high organizational resources.

**Discussion:**

Following the segmentation of the addressees, organizations might be supported in identifying needs and areas for prevention and health promotion. Interventions can be optimally developed, tailored, and coordinated through a deeper understanding of job demands and resources. Especially employees with low socioeconomic status and high job demands might profit from an addressee-orientated approach based on resource profiles. For example, employees obtain an overview of their occupational resource profile to recognize the development potential for safe and healthy behavior at work. Follow-up research should be used to examine how this feedback to employers and employees is implemented and how it affects the sustainability of tailored interventions.

## 1. Introduction

Occupational health management (OHM) supports employers and employees on both an individual and an organizational level (Hartung et al., 2021). However, OHM interventions are often offered in a generalized manner to all employees, and person-oriented approaches are difficult to implement. In order to use OHM interventions sustainably and effectively, it is important to adequately assess employers' and employees' demands and resources, allowing interventions to be optimally coordinated. Precision prevention (Gambhir et al., 2018) attempts to address the needs and requirements of individuals and reach them in an addressee-oriented manner. In contrast to a one-size-fits-all approach, where one intervention is designed for all employees, addressee orientation considers the individual differences and characteristics of individuals as well as groups of individuals (von Hippel and Tippelt, 2010). This creates a more precise data basis in the need assessment to subsequently develop and offer tailored interventions. Until now, an addressee-oriented approach has only, in a limited fashion, been taken into account in OHM (Viana et al., 2021). The precision health model (Gambhir et al., 2018) helps to modify OHM to include an addressee-oriented approach through the steps of health monitoring, data analytics, and tailored interventions. In the context of monitoring, it is relevant to generate comprehensive occupational health data from employees and companies. In data analytics, one possibility of addressee orientation is the segmentation of employers and employees (Hawkins et al., 2008). People with similar constellations regarding work-related health indicators are identified in subgroups within the entirety of the employee pool. Segmentation processes bundle groups of people, and individuals are grouped together by similar characteristics profiles (Rabel et al., 2019). In this study, we provide a more detailed insight into these groups by segmenting the employers and employees based on personal and perceived organizational resources. This is a starting point for tailored interventions, within needs and demands, or resources can be addressed and modified.

To address domain specificity, work-related health data are necessary (Gillman and Hammond, 2016). The job demands-resources model (Bakker and Demerouti, 2007) is used as a theoretical basis, combining two aspects of work-related health. The work context can be health-damaging due to a health impairment process (Fujishiro et al., 2010) and health-promoting due to a motivational process (Bertazzi, 2010; Demerouti and Bakker, 2011). Within the health impairment process, job demands deplete employees' resources (Bakker and Demerouti, 2007), which can lead to negative consequences such as increased stress levels or a decline in wellbeing. Specific psychological or organizational job demands are, for example, high workload, extra work, or overtime. Physical job demands are associated with lifting heavy weight loads or working under precarious environmental conditions such as cold, heat, humidity, or drafts. Physical job demands are relatively rigid and given, so employees can only influence them to a limited extent—however, employees adjust to and deal with environmental conditions. Work-related resources can thus modify the effect and impact of stressors on strain (Lohmann-Haislah, 2012) and, if necessary, buffer them in the case of negative effects (Bakker et al., 2003). Both personal resources and perceived organizational resources play a role in having positive effects on the health of individuals (Bakker and Demerouti, 2007; Roczniewska et al., 2022) and the productivity of organizations (Nielsen et al., 2017).

In the process of data analytics, we use job resources for the segmentation approach that gives a holistic picture and includes both individual's and organization's views. Then, we describe the profiles based on job demands and personal factors. Nevertheless, even within a holistic approach, it is important to focus on selected key indicators of resources and health. Thus, by reducing complexity, a middle ground between a one-size-fits-all approach and atomistic segmentation for tailored interventions becomes feasible (Kreuter et al., 1999; Hawkins et al., 2008). Personal occupational resources are included in our segmentation approach in terms of occupational health literacy (Friedrich et al., 2023) and occupational self-efficacy (Schyns and von Collani, 2002). Both constructs constitute factors in the work context that can be individually acquired, maintained, and strengthened. Factors related more to organizational processes and the work environment include job decision latitude (Nübling, 2005; Nübling and Hasselhorn, 2010) and opportunities to participate in health at work (Williamson, 2014).

### 1.1. Personal resources at the workplace

In recent years, health literacy in general, specifically in the workplace, has been much more present in the minds of individuals and in society (Schaeffer et al., 2021). Occupational health literacy comprises competencies with a knowledge- and skill-based approach to health and willingness to change and take responsibility for health (Friedrich et al., 2022). Knowledge about one's own occupational health literacy is helpful to cope with health challenges at work. When people know how to behave healthily in the work context, they use their knowledge and skills at work to cope with health demands and challenges. Getting active for one's own health benefits and the health of others at the workplace could be seen as prosocial motivation (Grant and Berg, 2011), which in turn could have positive impacts on the organization.

Occupational self-efficacy could predict the abilities and behaviors of an individual to fulfill work-related tasks (Felfe and Schyns, 2006), also known as the belief in occupational capability (Schyns, 2004; Peng et al., 2021). As one part of psychological capital (Luthans et al., 2007), self-efficacy is relatively malleable, acting as a leverage point in OHM interventions in improving health situations in organizations (Toor and Ofori, 2008; Avey et al., 2009). Furthermore, correlations between health literacy and self-efficacy were shown in many clinical studies (Xu et al., 2018) as well as for the domain-specific scales in the occupational context (Azizi et al., 2019). When people are able to use their potential and strengths at work and build and maintain their occupational health literacy and occupational self-efficacy, opportunities for participation in health at work or employees' influence on their workplace emerge.

### 1.2. Perceived organizational resources

Job decision latitude primarily encompasses the degree of autonomy with which decisions can be made about work content and the manner in which the activity is performed (Hackman and Oldham, 1975; Karasek, 1979). Job decision latitude is associated with employee health concerning physiological and strain symptoms (Elovainio et al., 2004). Moreover, correlations between the lack of influence at work and health impairments are evident (Elovainio et al., 2001; Kivimäki et al., 2019). By increasing job decision latitude when appropriate, negative physiological and psychological consequences for the employees can be buffered (Stahl and Hauger, 1994), and organizations can reconsider their workplace design.

The active involvement of employees in decision-making processes and activities related to health in the company forms an effective facet of the organizational climate. Participative climate influences work-related attitudes and behaviors (Tesluk et al., 1999). The perception of the employees concerning participation possibilities and the instrumental facet concerning the financial possibilities for health are decisive in creating and establishing health-promoting structures within a company (Kuenzi and Schminke, 2009). A positive correlation between job decision latitude and participation has been established (Höge, 2005). Furthermore, there exists an indirect effect of possibilities for participation through perceived organizational justice on psychophysical health (Höge, 2005). All in all, participation in health at work and the involvement of employees can be used to design healthy workplace interventions and are important factors in their success (Grawitch et al., 2009).

### 1.3. Aims of the present study

This study aimed to find and interpret occupational resource profiles with a special focus on health at work in small- and medium-sized enterprises (SMEs). In Germany, most of the companies are SMEs, and OHM has not yet been implemented systematically or in an addressee-oriented manner. Existing approaches cannot easily be transferred from large companies to SMEs. Therefore, the focus of the joint research project was on SMEs. The profiles are based on individual (occupational health literacy and self-efficacy) and perceived organizational resources (participation in health at work and job decision latitude). An occupational resource profile considers individual and organizational factors in the workplace that help employers and employees work in a sustainable healthy manner. The resources can include individual competencies or skills as well as organizational framework conditions for a healthy way of working. Occupational resource profiles thus summarize personal experiences, competencies, and framework conditions at work and provide information about the manifestation and level of work- and health-related patterns. With an interpretation and knowledge of profiles, the needs of employees can be better assessed in OHM and subsequently, interventions can be developed, matched, and tailored to the needs. First, we explore with cluster analysis, which profile solution fits the data best. Second, we use latent profile analysis to confirm the resource profiles found. Third, we use physiological and psychological job demands, hierarchy level, and socioeconomic status to describe the resource profiles for further interpretations for practice.

## 2. Methods

### 2.1. Sample and data collection

In total, 831 employers and employees in different SMEs in Germany were surveyed in a cross-sectional study through an external polling agency with computer-assisted telephone interviews. The inclusion criteria were working people in an SME with < 249 employees over the age of 18 years. Data collection took place from December 2020 to May 2021. Three subjects were excluded due to missing values, lack of concentration, and language barriers. The characteristics of the sample with *n* = 828 participants can be found in Table 1. The socioeconomic status (SES) of the participants was in the third quintile compared to the German Health Interview and Examination Survey for Adults (Lampert et al., 2013).

**Table 1:** Sample characteristics with number of participants (*n*), mean (*M*), standard deviation *(SD)*, minimum *(Min)*, and maximum *(Max)*.

**Characteristics**	**Participants**
	***n*** = **828**
**Gender**	
Female	53.70%
Male	45.00%
Diverse	1.20%
**Age**	
*M (SD)*	41.5 (12.2)
*(Min, Max)*	(18, 72)
**Hierarchy**	
Low: no personnel responsibility	72.00%
Medium: employee with personnel responsibility	17.40%
High: employer or owner	10.60%
**Socioeconomic status**	
*M (SD)*	11.1 (4.2)
*(Min, Max)*	(2, 21)
Low	26.20%
Medium	34.60%
High	39.10%
**Sectors**	
Trade/hospitality/transport	23.90%
Manufacturing industry	22.20%
Service sectors	35.00%
Administration and research	18.00%
Agriculture and forestry	0.70%

### 2.2. Measures of occupational resources for segmentation

#### 2.2.1. Occupational health literacy

The scale for assessing occupational health literacy contains 12 items. In the validation study of the occupational health literacy scale, factor analysis revealed a division into eight items for a knowledge- and skill-based approach to health and four items for willingness to change and take responsibility for health (Friedrich et al., 2023). The factor knowledge- and skill-based approach to health was measured on a scale from 1 (very difficult) to 4 (very easy). Example items were “In your opinion, how easy or difficult is it … to find information on safety and health at work in simple language?” (OHL 1) or “… to actively implement solutions when work situations are stressful for health reasons?” (OHL 4). The factor of willingness to change and take responsibility for health was captured on a scale from 1 (strongly disagree) to 4 (strongly agree). An example item was “I am very conscious of taking an active role in promoting health in the workplace” (OHL 12). Higher scores indicate a greater knowledge- and skill-based approach to health or willingness to change and take responsibility for health. The two factors were included individually as mean scores in the calculations.

#### 2.2.2. Occupational self-efficacy

Eight items were used to assess occupational self-efficacy utilizing the short version of the occupational self-efficacy scale (Schyns and von Collani, 2002) from 1 (strongly disagree) to 5 (strongly agree). Higher scores indicated higher occupational self-efficacy. For data analyses, a mean score of all eight occupational self-efficacy items (e.g., “I feel prepared to meet most of the demands in my job”, OSE 5) was built.

#### 2.2.3. Job decision latitude (influence and latitude at work)

Six items captured the possibilities for influencing the workplace inspired by the dimension “influence at work” of the English third version of the Copenhagen Psychosocial Questionnaire (COPSOQ) (Nübling, 2005; Nübling and Hasselhorn, 2010; Burr et al., 2019). The scope of action at work on decisions (e.g., “Do you have a large degree of influence on the decisions concerning your work?”, JDL 1), quantity and quality of work (e.g., “Do you have any influence on what you do at work?”, JDL 5 or “Do you have any influence on how you do your work?”, JDL 6), and how quickly and with whom the work is done built the mean score on job decision latitude. On a scale from 1 (never) to 5 (always), higher scores indicated greater job decision latitude.

#### 2.2.4. Participation in health at work (PAR)

A self-constructed scale with four items encompassing the facets of participation in health at work was validated. The items were “I can have a say in matters related to my health at work.” (PAR 1); “It is important to be open to suggestions when it comes to health at work.” (PAR 2); “In our company, there are many opportunities to help shape a healthy work situation.” (PAR 3); and “Suggestions on workplace health are very welcome in our company.” (PAR 4). Higher values on a scale from 1 (strongly disagree) to 5 (strongly agree) indicated a greater possibility of participation in health at one's own workplace. Cronbach's α was 0.79, which is acceptable.

### 2.3. Measures for the description of the segments

#### 2.3.1. Psychological job demands

To adequately describe the specific segments, job demands were elicited through items in accordance with the COPSOQ (Nübling, 2005; Nübling and Hasselhorn, 2010; Burr et al., 2019). We used four items from the German section, namely work pace, quantitative demands, control over working time, and demands for hiding emotions. Response categories ranged from 1 (never) to 5 (always).

#### 2.3.2. Physiological job demands

For a broader view, we added four items on physical job demands from the COPSOQ on the frequency of carrying heavy loads, working in noise, or other adverse environmental conditions. We supplemented a self-constructed item on working in unusual postures (“How often do you have to work bent over, squatting, kneeling, lying down?”). Following the original literature, we built a standard score from 0 to 100 for both job demands. Afterward, we constructed three categories for a more comprehensive profile description, using lower than 37.5 points for low demands, more than 37.5–62.5 points for medium, and more than 62.5 points for high job demands.

#### 2.3.3. Hierarchy

The level of hierarchy indicated personnel responsibility at three levels. Employees with the lowest level had no personnel responsibility. People with a medium hierarchy level were employed and had responsibility for at least one person. In the highest hierarchy level, there were employers or people without their own supervisors.

#### 2.3.4. Socioeconomic status

SES included school and professional qualification, professional status, and household net income (Lampert et al., 2013). In the original literature, three categories, such as low (0–7.7), medium (7.8–14.1), and high (14.2–21) SES scores, were built (Lampert et al., 2013). With 28% missing values on net income, we followed previous literature (Lampert et al., 2013; Frick and Grabka, 2014) and calculated the SES score with imputed missing values.

### 2.4. Statistical procedure and analyses

R statistics version 4.1.3 was used for data analyses. We first grounded the resources incorporating new items, such as participation in health at work, in our measurement model and factor structure. The variables were factor-analyzed to determine which items were loaded on which scores or which items could be eliminated if necessary. We then performed a segmentation with cluster analyses based on the factor scores. Missing values were imputed with a weighted average of the *k*-nearest neighbor algorithm (*k* = 10). For the comparability of variables, scores were standardized. In the first step, a hierarchical cluster analysis with single-linkage procedures and a Euclidean distance measure was calculated. Outliers with extreme values were identified and eliminated. Then, scores were re-formed with the adjusted dataset, and a hierarchical cluster analysis was performed using Ward's method with a Euclidean distance measure. Using a graphical scree plot, dendrogram, and history of the residual sum of squares (RSS) as well as the increase in RSS with further fusion, a favoring number of clusters were determined using Ward's procedure. Thus, a cluster center analysis with a *k*-means algorithm was performed to optimize the cluster solution.

In the second step, a latent profile analysis using R package mclust (Scrucca et al., 2016) was performed to verify the structure and find a solution. Then, scales were built and centered. Bayesian information criterion (BIC) and integrated completed likelihood (ICL) were used to decide on the best solution. ICL is seen as more robust than BIC (Biernacki et al., 2000). Smaller values indicated a better model fit.

Finally, characteristic values of the formal description of the cluster solution helped in interpreting the profiles and finding a content description. Physical and psychological job demands were combined with the profile solutions to provide theoretical support for a segment description and to see which resource profiles were associated with specific demands or socioeconomic variables.

Latent profile analysis is a categorical latent variable modeling approach that focuses on identifying latent subpopulations within a population based on a certain set of variables. There are no firmly established methods for calculating *a priori* statistical power for latent profile analyses (Tein et al., 2013). *A priori* case number planning for independent *t*-tests was performed. The power with this number of cases (significance level 5% two-sided, power 80%) was already large enough to detect even small effect sizes of 0.20.

## 3. Results

### 3.1. Pre-analyses: exploratory factor analyses

A parallel analysis suggested six factors and five components of the initial 30 items. An exploratory factor analysis with principal axis method and oblimin rotation showed that for six factors, in addition to the expectancy-compliant constructs, two items of job decision latitude (“Do you have any influence on what you do at work?”, JDL 5 and “Do you have any influence on how you do your work?”, JDL 6) and one item of participation in health at work (“It is important to be open to suggestions when it comes to health at work”, PAR 2) were loaded on a common factor. In addition, it appeared that this item on participation in health at work captured openness to workplace health suggestions rather than directly perceived participation opportunities in the company. Factor loading was relatively low at 0.46. Therefore, one item was excluded from the analyses, resulting in three items for participation in health at work. Afterward, exploratory factor analyses revealed acceptable internal consistencies and factor structures of the constructs with eight final items for the knowledge- and skill-based approach to health (Cronbach's α = 0.87), four items for willingness to change and take responsibility for health (α = 0.76), eight items for occupational self-efficacy (α = 0.86), six items for job decision latitude (α = 0.83), and three items for participation in health at work (α = 0.82).

### 3.2. Missing values and outliers for cluster analyses

Overall, the proportion of missing values in the data set of *n* = 828 participants was small, and 34 individuals had a missing value on at least one item. Frequently, some individuals showed up to five missing values, so listwise case exclusion would have resulted in a sample of *n* = 794. For this reason, *k*-nearest neighbor imputation (*k* = 10) of the missing data was performed, which was used in similar contexts and was able to provide good results for different distributions of missing data (Liao et al., 2014; Cleophas and Zwinderman, 2016; Aktaş et al., 2019). In *k*-nearest neighbor imputation, the mean values of the neighboring values are formed and assigned to the missing value (von der Hude, 2020). This provided a complete dataset with which further analyses can be conducted.

Supplementary material provides an overview of the hierarchical cluster analysis using single-linkage methods and a Euclidean distance measure. There was a disproportionate increase in the fusion value at step 823 from 6 to 5 clusters, suggesting the need to further inspect these five individuals. In particular, the cluster centers on occupational self-efficacy of these five subjects showed extreme values with *z*-scores close to −3 and −4. In addition, one individual showed outliers on the knowledge- and skill-based approach to health and an inconsistent pattern on willingness to change and take responsibility for health. For these reasons, five outliers were excluded, resulting in *n* = 823 for further analyses. Table 2 provides an overview of the descriptive statistics of the mean scores.

**Table 2:** Descriptive statistics of the used occupational resources.

	***M (SD)***	***Mdn***	***Min–Max***	***S***	***K***	**SE**	**1**	**2**	**3**	**4**
1. Knowledge- and skill-based approach to health	3.0 (0.6)	3.0	1.0–4.0	−0.10	−0.31	0.02				
2. Willingness and responsibility for occupational health	3.3 (0.6)	3.2	1.2–4.0	−0.51	−0.66	0.02	0.28^**^ (0.22, 0.34)			
3. Occupational self-efficacy	4.0 (0.6)	4.0	1.2–5.0	−0.61	0.79	0.02	0.47^**^ (0.41, 0.52)	0.34^**^ (0.28, 0.40)		
4. Job decision latitude	3.5 (0.9)	3.5	1.0–5.0	−0.10	−0.55	0.03	0.27^**^ (0.20, 0.33)	0.20^**^ (0.14, 0.27)	0.38^**^ (0.30, 0.42)	
5. Participation in health at work	3.8 (1.0)	4.0	1.0–5.0	−0.51	−0.32	0.03	0.45^**^ (0.39, 0.50)	0.33^**^ (0.27, 0.39)	0.40^**^ (0.34, 0.45)	0.50^**^ (0.45, 0.55)

*M*, Means; *SD*, standard deviations; *Mdn*, median; *S*, skewness; *K*, kurtosis; SE, standard errors; and correlations (*r*) with confidence intervals of the mean scores with *n* = 823. ^**^p < 0.01.

### 3.3. Determining the number of clusters for segmentation

A cluster analysis using Ward's method provided clues to a cluster solution. Individuals (*n* = 823) were assigned to clusters in a stepwise manner so that the variance within groups remained as small as possible. In the scree plot, an indication of six clusters could be interpreted by the visual elbow criterion. The course of the RSS showed a higher increase from seven to six clusters and from six to five clusters, respectively. This could be taken as an indication that no more than six clusters should be extracted.

The *k*-means cluster analysis outputs further measures of the quality of the cluster solution, which are summarized in Table 3. The explained dispersion η^2^_c_ showed that at a cluster number of five, more than half of the variance was explained by the clusters. A 4% increase in variance was the greatest at the transition from cluster five to six compared to the other transitions starting at six clusters. Moreover, up to a number of six clusters, relatively evenly distributed clusters were formed. Only from a cluster number of seven, clusters of smaller size (< 10% of the total cases or < 82 individuals) were formed, which may give an indication of good stability. In summary, a 6-cluster solution was chosen.

**Table 3:** Summary of the cluster analysis.

**Cluster**	**Scree plot**	**RSS increase**	**BSS/TSS η^2^_c_**	**PRE_c_**	***n*C < 10%**
2		350	33.0%	0.33	0/2
3		282	41.8%	0.13	0/3
4		203	48.5%	0.12	0/4
5		173	53.5%	0.10	0/5
6	*x*	113	57.5%	0.09	0/6
7		59	60.3%	0.07	1/7
8		93	62.6%	0.06	3/8

Scree plot: visual elbow method of the residual sum of squares against the number of clusters; RSS, residual sum of squares; BSS, between-cluster sum of squares; TSS, total sum of squares; PRE_c_, proportionate reduction of error. *n*C, number of clusters < 10% of the total cases (*n* = 823).

### 3.4. Main results: latent profile analyses

We excluded the same outliers as determined in the hierarchical cluster analysis for better comparability. A latent profile analysis with ellipsoidal, equal shape, orientation (VEE), and *n* = 823 showed the smallest BIC for five profiles, closely followed by a model with VEE for six profiles (BIC = −10,740). However, a model with spherical covariances and equal volume (EII) represents the same procedure as that of Ward's method for hierarchical clustering (Scrucca and Raftery, 2015; Scrucca et al., 2016). For the comparability of the two methods, we used a model with EII, whose BIC was only marginally higher (BIC = −10,891). Bootstrap Likelihood Ratio Tests (BLRT) were significant for less than nine profiles, indicating that no more than eight profiles were suitable (see Table 4). In a model with EII, the BIC showed the lowest values for eight profiles, followed by seven and six profiles. Due to the assumption that the BIC overestimated the number of profiles when the model fit was not that good and well-separated profiles were favored (Biernacki et al., 2000), we used the ICL value, which was the smallest for six profiles. Regarding the number of profiles, due to a parsimonious solution, we examined the allocation probabilities per profile. In a further descriptive step, only individuals with an allocation probability over 0.80 to their profile were considered (Collins and Lanza, 2010). The model with six profiles had a good assignment with 58.2% (*n* = 479) individuals with allocation probability over 0.80 in comparison to the model with eight profiles with only 39.2% (*n* = 323) individuals. Furthermore, in two profiles of the 8-profile solution, no individual had an allocation probability over 0.80, while the parameter means of the other profiles stayed almost the same. There was a risk of instability in parts of the profiles of the 8-profile solution. Two profile sizes were smaller than 10% (*n* = 42 and *n* = 61), followed by two profile sizes with 11.5% (*n* = 95) and 11.7% (*n* = 96). Therefore, from a descriptive point of view, we selected the six-profile solution with only one profile size smaller than 10% (*n* = 45). The parameter means of the profiles were in the same direction as shown in the cluster analysis, indicating the same results.

**Table 4:** Fit indices and model comparisons of the latent profile analysis with allocation probabilities.

								**Allocation probabilities per profile (in %)**							
**Profile**	**LL**	**BIC**	Δ**BIC**	**ICL**	**LRTS**	**BLRT** ***p***	***n*****P**<**10%**	**1**	**2**	**3**	**4**	**5**	**6**	**7**	**8**
2	−5,494	−11,068	−645	−11,236	687	0.001	0	90.1	91.8	–	–	–	–	–	–
3	−5,443	−11,007	−61	−11,335	104	0.001	0	81.0	86.8	84.1	–	–	–	–	–
4	−5,415	−10,992	−15	−11,423	53	0.001	0	69.1	74.0	86.2	85.6	–	–	–	–
5	−5,385	−10,971	−21	−11,449	61	0.001	0	69.7	71.1	85.3	87.0	69.0	–	–	–
6	−5,325	−10,891	−80	−11,312	116	0.001	1	75.6	73.2	86.6	86.5	73.8	83.2	–	–
7	−5,302	−10,885	−6	−11,397	46	0.001	1	72.5	73.0	84.2	86.0	72.0	68.1	83.2	–
8	−5,276	−10,875	−10	−11,390	49	0.001	2	71.7	73.6	84.3	83.0	74.9	73.9	69.3	84.1
9	−5,276	−10,896	21	−11,447	18	0.023	3	–	–	–	–	–	–	–	–

LL, Log-likelihood; BIC, Bayesian information criterion; ICL, integrated completed likelihood; LRTS, likelihood ratio test statistic; BLRT, bootstrap likelihood ratio test; *n*P < 10% = number of profiles with < 10% of the individuals.

### 3.5. Interpretation of the six-profile solution

The interpretation of the profiles was based on statistical characteristics and content knowledge of the constructs. Content characterizations and interpretations were made by outputting the parameter means of the latent profile analysis for the six-profile solution. Table 5 shows the profile characteristics. Assessments of the interpretation of content indicated that there is one profile with consistently above-average values (P3), which is contrasted by one profile with below-average means (P4). Four profiles had differentiated expressions with, on the one hand, above-average organizational resources with average personal resources (P5) and, on the other hand, above-average personal resources with (below) average organizational resources (P2). A profile had below-average organizational resources and below-average knowledge- and skill-based approach to health, contrasted by above-average willingness to change and take responsibility for health (P6). Individuals in another profile had both slightly above-average organizational resources such as job decision latitude and below-average personal resources in the form of willingness to change and take responsibility for health (P1).

**Table 5:** Characteristics of the six profiles with profile size (in percentage).

**Profile**	**Characteristics**
**Number of participants (%)**	
P1 100 (12.2%)	Low motivated profile with above-average organizational resources • Below-average personal resource: willingness and responsibility for health • Average personal resource: knowledge- and skill-based approach to health • Slightly above-average organizational and personal resources: job decision latitude and participation in health and occupational self-efficacy
P2 112 (13.6%)	Low job decision latitude with above-average personal resources • Below-average organizational resource: job decision latitude • Average organizational resource: participation in health at work • Above-average personal resources: knowledge- and skill-based approach to health and willingness and responsibility for health and occupational self-efficacy
P3 162 (19.7%)	Above-average profile • Highest values on all resources • Especially knowledge- and skill-based approach to health with the highest values
P4 189 (23.0%)	Below-average profile • Lowest values on all resources • Especially occupational self-efficacy with the lowest values
P5 215 (26.1%)	Nearly average profile, higher organizational resources, and motivation • Average personal resources: knowledge- and skill-based approach to health and occupational self-efficacy • Above-average organizational resources: job decision latitude and participation in health • Above-average personal resource: willingness and responsibility for health
P6 45 (5.5%)	Lowest participation in health but above-average motivation • Below-average organizational resources: job decision latitude and participation in health • Below-average personal resources: knowledge- and skill-based approach to health • Average personal resource: occupational self-efficacy • Above-average personal resource: willingness and responsibility for health

Exact profile centers can be made available upon reasonable request to the authors.

### 3.6. Segmentation description

For a profile description, we analyzed the relationships between job demands as well as hierarchy levels, SES levels, and resource profiles. Fisher's exact tests indicated statistically significant differences between the profiles and each variable (*p* < 0.001). Table 6 shows the distributions of the six profiles for the demand categories. For low psychological and physiological job demands, profiles 3 and 5 occurred most frequently. For high job demands, profiles 1, 4, and 6 were significantly overrepresented.

**Table 6:** Frequencies (and percentage) of the participants in the profiles distributed by demands.

		**Psychological job demands** ***n*** = **823**			**Physiological job demands** ***n*** = **795**		
**Profile**	**Label**	**Low**	**Medium**	**High**	**Low**	**Medium**	**High**
1	Low willingness and responsibility for occupational health, high organizational resources	44 (48.4%)	34 (37.4%)	13 (14.3%)	72 (72.0%)	14 (14.0%)	14 (14.0%)
2	Low job decision latitude, high personal resources	57 (52.3%)	40 (36.7%)	12 (11.0%)	84 (75.0%)	17 (15.2%)	11 (9.8%)
3	Above-average resources	93 (58.5%)	53 (33.3%)	13 (8.2%)	126 (77.8%)	27 (16.7%)	9 (5.6%)
4	Below-average resources	69 (37.7%)	74 (40.4%)	40 (21.9%)	133 (70.4%)	38 (20.1%)	18 (9.5%)
5	Average with high organizational resources	112 (53.6%)	78 (37.3%)	19 (9.1%)	173 (80.5%)	32 (14.9%)	10 (4.7%)
6	High willingness and responsibility for occupational health, low organizational resources	15 (34.1%)	19 (43.2%)	10 (22.7%)	25 (55.6%)	13 (28.9%)	7 (15.6%)
	Percentages per level	49.1%	37.5%	13.5%	74.5%	17.1%	8.4%

Dark-colored cells represent the most frequent participants in the levels compared to the whole sample. Gray-colored cells indicate the second most frequencies.

The distributions for hierarchy and SES can be seen in Table 7, where most people with low hierarchy levels appeared in profiles 4 and 6 and with high hierarchy levels in profiles 3 and 5. For low SES, the participants were most frequently represented in the below-average profile 4 and profile 2 with low job decision latitude. Profile 1 and 3 with above-average values were the most common for participants with high SES.

**Table 7:** Frequencies (and percentage) of the participants in the profiles distributed by hierarchy and SES levels.

		**Hierarchy** ***n*** = **823**			**Socioeconomic status** ***n*** = **823**		
**Profile**	**Label**	**Low**	**Medium**	**High**	**Low**	**Medium**	**High**
1	Low willingness and responsibility for occupational health, high organizational resources	62 (62.0%)	24 (24.0%)	14 (14.0%)	11 (11.0%)	54 (54.0%)	35 (35.0%)
2	Low job decision latitude, high personal resources	97 (86.6%)	15 (13.4%)	0 (0.0%)	38 (33.9%)	61 (54.5%)	13 (11.6%)
3	Above-average resources	91 (56.2%)	39 (24.1%)	32 (19.8%)	26 (16.0%)	91 (56.2%)	45 (27.8%)
4	Below-average resources	178 (94.2%)	9 (4.8%)	2 (1.1%)	82 (43.3%)	90 (47.6%)	17 (9.0%)
5	Average with high organizational resources	126 (58.6%)	51 (27.7%)	38 (17.7%)	48 (22.3%)	125 (58.1%)	42 (19.5%)
6	High willingness and responsibility for occupational health, low organizational resources	40 (88.9%)	4 (8.9%)	1 (2.2%)	11 (24.4%)	31 (68.9%)	3 (6.7%)
	Percentages per level	72.2%	17.3%	10.6%	26.2%	54.9%	18.8%

Dark-colored cells represent the most frequent participants in the levels compared to the whole sample. Gray-colored cells indicate the second most frequencies.

## 4. Discussion

The present study characterizes different occupational resource profiles in a German setting of SMEs. Occupational health literacy (with a factor on knowledge- and skill-based approach to health and a factor on willingness and responsibility for health) and occupational self-efficacy tend to be seen as personal resources. Job decision latitude and participation possibilities in health are rather seen as organizational resources. The above-average profile 3 and the below-average profile 4 build two poles regarding occupational resources. The other profiles of the six-profile solution are in between and have quite different characteristics. A closer look at these four profiles shows that the individual and perceived organizational resources are contrasting. People in the smallest profile 6 have above-average motivational resources but below-average organizational resources such as participation in health at work or job decision latitude. The same pattern occurs for profile 2 with the difference that people of this profile have an above-average individual knowledge- and skill-based approach to health and average occupational self-efficacy. While having slightly above-average perceived organizational resources in profile 1, the motivational part of health literacy is below-average. Profile 5 has the closest values to the average with slightly above-average organizational resources and willingness to take responsibility for health.

Our hybrid approach focuses on segmenting employers and employees by health resources and on comparing the job demands, socioeconomic status, and hierarchy levels for a better understanding of profile description (Jenkins et al., 2021). Other studies often use sociodemographic factors (Boslaugh et al., 2005; Jenkins et al., 2021) for segmentation analyses. Sociodemographic factors such as age and gender are determined, contrasting to resources at work that can be learned or changed and are under the control of individuals or organizations. Furthermore, the SES index gives a deeper insight into the reality and backgrounds of the people and can be used to identify addressees for interventions (Lampert et al., 2013). Based on these findings, a more addressee-oriented view can be considered to help provide tailored interventions for the utilization of employers, occupational physicians, or human resource managers in precision prevention, as well as best fitting measures for employees.

When describing the profiles, people with a low socioeconomic status are most frequently in the below-average profile 4 or in profile 2, with low job decision latitude but high personal resources. For employees with low SES, tailored interventions could be leveraged for support, allowing for increased possibilities to design a workplace aligned with the employee's personal knowledge and skills. People with a high SES are most frequently in profile 1 (limited only by the motivational resource) and in the above-average profile 3. In interventions, the motivation of these employees can be addressed, and they can act as multipliers for health in their workplaces.

Individuals with high levels of hierarchy are represented more in profiles with higher organizational resources. Employees in the lowest hierarchy level are mostly distributed into profiles 2, 4, and 6, which are the profiles with below-average perceived organizational resources. Therefore, the hierarchy level makes a decisive point on resources in the company. Employers (in our study, 10.6% of the participants) might have a higher impact on the organizational culture or climate than employees. Nevertheless, for high hierarchy levels, a profile with a slightly above-average willingness and responsibility for health occurred second most. Building individual knowledge and skills on health and maintaining the willingness to take on responsibility for health—especially among employers and employees with high organizational resources such as influence or participation in health at work—can be a promising intervention to change an organization into a healthy workplace.

For physiological and psychological job demands, nearly the same pattern occurred: Employers and employees with low job demands emerged the most in the above-average profile 3. Furthermore, people with high levels of psychological demands are overrepresented in the below-average profile 4 and profile 6 with high motivation and limited organizational resources. One interpretation of these data is that employers and employees with high psychological job demands want to change their situation and take responsibility for their health at work. In contrast, people with high physiological job demands are also in the low motivational resources profile 1 while having slightly above-average organizational resources. This indicates that people with high physiological job demands either benefit from the organizational situation or have the possibility to make their workplace more conducive to health. In contrast, other people with high physiological demands see themselves as motivated and take responsibility for their own health. In our study, only a few participants reported high job demands, indicating that our sample had relatively adequate work situations regarding demands. However, we formed categories for the demands for better comprehensibility, which could distort the actual scale. Nevertheless, even people with medium demands are most frequently in the below-average profile 4 and the below-average organizational resources profile 6 with a high motivational factor. A previous study on job demands-resources profiles (Van den Broeck et al., 2012) found four profiles with demanding, resourceful, poor, and rich jobs. As mentioned in the job demands-resources model, demanding jobs were associated with lower resources (Van den Broeck et al., 2012), which can be compared with our results: People in demanding jobs most frequently have below-average resources. Job demands cannot be changed easily, but often employees in demanding jobs are motivated to change their situation and perceptions, which can consequentially lead to an increase in resources and, in the long term, wellbeing (Tims et al., 2013).

To generalize, there is a dynamic exchange, and individuals are in between job demands and job resources and have to be and remain healthy in the long term. An interplay of personal and organizational factors can help the individual and the organization. On the one hand, it is important to move within the organization in such a way that one's own health is maintained and promoted, thus making the workplace health friendly. A previous study shows that when employees were more involved, an association with higher levels of organizational commitment and job satisfaction occurred (Cox et al., 2006). On the other hand, an organization benefits from healthy employees in the form of higher productivity or lower absenteeism (van der Voordt and Jensen, 2021). Additionally, individuals change the organizational structure and the view of health in the mission statement or organizational climate. These changes can, in turn, have a positive impact on employee perceptions (Mutonyi et al., 2022).

In terms of occupational health literacy, it is also important to communicate adequately about health. Health-literate organizations should provide a system where employees can act and behave in a healthy way (Bitzer and Schaefer, 2022). An understanding of individual resources in the workplace is as important as a view of organizational opportunities. To specify, a deeper understanding of the interplay between resources and demands not only on the individual but also on the various work environments (Nöcker, 2016) can lead to better communication and more coordinated interventions and actions (van Holland et al., 2018). The segmentation of employers and employees with occupational resource profiles and the consideration of job demands as well as socioeconomic factors are the starting points for tailored interventions and an addressee-oriented approach in companies.

### 4.1. Limitations

Due to the special focus of the diversity-sensitive research project, the sample was relatively diverse; however, with 47.5% migration background in participants, it is not representative of the whole workforce of Germany. Furthermore, employers and employees in several SMEs were investigated. While every SME is different, the results cannot be generalized easily to large companies or other countries (Champoux and Brun, 2003). In this study, heterogeneous industries were examined based on the Federal Statistical Office in Germany. Therefore, the sample includes heterogeneous companies that have different environmental conditions. Non-response may be higher for employees working in adverse conditions. Additionally, employees' wages and work history should be considered so that there are no potential biases related to health and wellbeing (Böckerman et al., 2012). We included socioeconomic status with household net income to account for this aspect. Looking at work history, which includes working in different sectors or different physical or psychological job demands, could give deeper insights and prevent biases. Furthermore, individuals can only report on their own experiences of health in the workplace. These statements are subjective and may be influenced by social desirability or other time-related factors. Data collection took place during the COVID-19 pandemic in Germany and covered a period of 6 months. Within this time, there was a lot of information on occupational safety and health that could also have an influence on perceived control or health literacy. These factors are experienced individually and captured by subjective assessments. However, they may reflect the perceived view of the organization. Actual organizational factors of health in the company such as absenteeism or other key performance indicators can help foster a more objective view.

Regarding the structure of the profiles, we found one relatively small profile with 5.5% of the sample size. In a profiling approach, this size is still defensible. With a larger sample, the evidence for and representativeness of the profiles could be increased. To confirm the latent structure, we chose a profiling model with a spherical, equal-volume shape. A latent profile analysis with this sample size could not definitively answer the question of how many subgroups really exist within the whole population. When adding new participants or variables, other profiles might emerge. Nevertheless, a model with an ellipsoidal equal shape and orientation also showed six profiles such that this structure could be confirmed in different approaches.

### 4.2. Implications and recommendations for research and practice

In future research and practice, OHM interventions of a company can be matched to the specific profiles. Addressee-oriented offers directly address employed people, resulting in a company's financial and time resources being saved. Furthermore, a personalized approach can motivate employees and strengthen their sense of personal responsibility. Empowerment helps to reveal employees' specific potential for further development in the area of health promotion and prevention. Longitudinal data would be interesting as this enables the study of the stability of profiles or changes in individuals. Additionally, occupational resource profiles might be used to evaluate the effectiveness of an intervention. Afterward, adaptations to the interventions can be made more specifically and precisely.

It was shown that health literacy and health are less pronounced among vulnerable groups and less educated employees (Berens et al., 2022). Under these circumstances, further development of OHM and occupational healthcare based on occupational resources such as health literacy and SES would be fruitful. Furthermore, it would be interesting to use the occupational resource profiles in other countries or contexts to compare the results and/or to understand relationships or differences. Particularly in the span of the COVID-19 pandemic, the forms of work have increasingly changed, for example, the observable shift to mobile work models. The occupational resource profiles help to improve the OHM of a company in an addressee-oriented fashion, facilitating the legal obligations in occupational safety and health.

## 5. Conclusion

Employers and employees bring their individuality and their own mental models (e.g., with life realities, health literacy, and self-efficacy) into the organizational system. In our analyses, by combining individual and organizational perspectives, a more comprehensive picture of the system, the individual, and the approach to OHM emerges. Optimally, the requirements in the work environment are better matched to the competencies of individuals so that personal developments in the organization are achieved (ÖPGK, 2019). In promoting health and looking at occupational resources, changes are possible on a personal level that can affect the organizational level and vice versa. We used a segmentation approach for addressee orientation and confirmed a six-profile solution. The occupational resource profiles provided a distribution of people with different individual and perceived organizational resources in the work environment. While combining the resource profiles with job demands, socioeconomic status, and hierarchy, we got a deeper insight into the interplay of job resources. In this fashion, the effect of resources on stress and strain can be used to promote individual health and change organizational conditions. By being assigned to a specific occupational resource profile, individuals can benefit and learn more about their resources, while organizations can simultaneously receive recommendations for tailored interventions.

## Data availability statement

The raw data supporting the conclusions of this article will be made available by the authors, without undue reservation.

## Ethics statement

The studies involving human participants were reviewed and approved by Ethics Commission of the Charité—Universitätsmedizin Berlin. Written informed consent was not provided because participants gave verbal informed consent in computer-assisted telephone interviews.

## Author contributions

JF was in charge of data collection, analyzed the data, conceptualized, and wrote the manuscript draft. GS and A-KM supported in the analyses, made substantial contributions, and critically revised the manuscript along with AT. GS, AT, and SV-M acquired funding for the joint research project. All authors read and approved the submitted version.
