# Supplementary material for: Occupational resource profiles for an addressee orientation in occupational health management: a segmentation analysis
Source: Front Psychol. 2023 Jul 20;14:1200798. doi: 10.3389/fpsyg.2023.1200798 (PMC10400086; doi:10.3389/fpsyg.2023.1200798)
Supplement: Supplementary file 1 [file Data_Sheet_1.docx]

Supplementary Material

Occupational resource profiles for an addressee orientation in occupational health management: A segmentation analysis

Julian Friedrich^*^, Anne-Kristin Münch, Ansgar Thiel, Susanne Voelter-Mahlknecht, Gorden Sudeck

*** Correspondence:**Julian Friedrich
julian.friedrich@uni-tuebingen.de

# Supplementary Data

# *Table 1. Course of the fusion values in the single-linkage analysis with Euclidean distance measure.*

| Step | Number of clusters | Fusion values | Growth |
| --- | --- | --- | --- |
| 814 | 14 | 1.545 | .000 |
| 815 | 13 | 1.565 | .020 |
| 816 | 12 | 1.587 | .022 |
| 817 | 11 | 1.622 | .035 |
| 818 | 10 | 1.637 | .015 |
| 819 | 9 | 1.729 | .092 |
| 820 | 8 | 1.842 | .113 |
| 821 | 7 | 1.880 | .038 |
| 822 | 6 | 1.902 | .022 |
| *823* | *5* | *2.097* | *.195* |
| 824 | 4 | 2.104 | .007 |
| 825 | 3 | 2.164 | .060 |
| 826 | 2 | 2.255 | .091 |
| 827 | 1 | 2.280 | .025 |

*Table 2. Cluster centers of the outliers at each step.*

| Step | Knowledge- and skill-based approach to health | Willingness and responsibility for occupational health | Occupational self-efficacy | Job decision latitude | Participation in health at work | |
| --- | --- | --- | --- | --- | --- | --- |
| 823 | -2.56 | -2.13 | *-3.094* | -2.71 | 1.3 |  |
| 824 | *-3.41* | 1.19 | 0.016 | 1.67 | 1.3 |  |
| 825 | 1.70 | -1.30 | *-3.678* | 1.30 | 1.3 |  |
| 826 | 1.70 | -0.47 | *-4.066* | 0.39 | 0.3 |  |
| 827 | 0.79 | 0.36 | *-4.261* | 1.30 | 1.3 |  |
